# Traditional Chinese exercise for COVID-19: A protocol for systematic review and meta-analysis

**DOI:** 10.1097/MD.0000000000023044

**Published:** 2020-11-06

**Authors:** Yuanyuan Duan, Mengran Xiong, Heping Wang, Xiaoyan Yao, Henyuan Liu, Guangxi Li

**Affiliations:** Guang ’anmen Hospital, China Academy of Traditional Chinese Medicine, Beijing, China.

**Keywords:** COVID-19, protocol, systematic review, traditional Chinese exercise

## Abstract

**Background:**

A new type of coronavirus (COVID-19), is spreading all over the world. Under the background of the comprehensive medical treatment and strict prevention and control in China, the number of discharged patients increased substantially. By the end of July, more than 80,000 patients had been cured and discharged from hospital in China. In order to effectively promote the full recovery of the patient's physical and mental functions and quality of life, gradually shift the emphasis of clinical work to convalescence therapy is very important, thus Chinese experts draw up Expert Consensus on Rehabilitation of Chinese Medicine for COVID-19. This systematic review and meta-analysis will assess studies of the effects of traditional Chinese exercise (TCE) for COVID-19 patients.

**Methods and analysis:**

We will search 6 English and 4 Chinese databases by 01, December 2020. After a series of screening, Randomized Clinic Trials (RCTs) will be included related to TCE for COVID-19. Two assessors will use the Cochrane bias risk assessment tool to assess the RCTs. Finally, the evidence grade of the results will be evaluated.

**Results:**

This study will provide a reliable evidence for the selection of TCE therapies for COVID-19.

**Conclusion:**

The results of this study will provide references for the selection of TCE treatment for COVID-19, and provide decision making references for clinical research.

**PROSPERO registration number:**

CRD42020179095.

## 1 Introduction

In December 2019, a cluster of pneumonia of unknown cause was reported in Wuhan, Hubei, China. On February 11, 2020, the world health organization officially named the disease caused by the new coronavirus as coronavirus disease 2019 (COVID-19).^[1]^ Under the background of the comprehensive medical treatment in China, the epidemic trend of COVID-19 gradually slowed down, and the number of discharged patients increased substantially. By the end of July, more than 80,000 patients had been cured and discharged from hospital in China. During the period of the treatment, we found that the patients with COVID-19 suffer from varying degrees of respiratory, physical, and psychological dysfunction,^[2]^ the isolation treatment of light and ordinary patients in the “Fangcang shelter hospitals” is prone to cause psychological problems such as tension and anxiety,^[3,4]^ and the treatment of long-term sickbed patients is easy to cause some complications such as pressure ulcer, deep venous thrombosis, muscle atrophy.^[5–7]^ The patient discharged from the hospital may still have symptoms such as fatigue, cough, poor mental state, and other symptoms after the nucleic acid turn negative.^[8–10]^ The patients may have varying degrees of lung function damage, interstitial pneumonia changes, and even the possibility of pulmonary fibrosis, and the risk of re-infection is not excluded.^[11–13]^ In order to effectively alleviate the symptoms, promote the recovery of the immune system and cardiopulmonary function, relieve anxiety, improve the patients’ physical and mental function, life quality, and comprehensive recovery of social participation, it's important to gradually shift the emphasis of clinical work to COVID-19 convalescence therapy, especially pulmonary rehabilitation.

Pulmonary rehabilitation is an individualized rehabilitation therapy for patients with chronic pulmonary disease after detailed evaluation. Focus on athletic training and integrated interventions such as psychological and nutritional support, education, and behavioral change.^[14]^ Clinical studies have shown that traditional Chinese medicine (TCM) related pulmonary rehabilitation techniques can provide beneficial support for respiratory diseases, relieve associated symptoms, and improve the overall quality of life. High-quality meta-analysis showed that shadowboxing and Baduanjin Exercise intervention in COPD could improve mobility, lung function, and quality of life with no adverse events, and, they are better than conventional treatment.^[15–17]^

Therefore, the Specialized Committee of Pulmonary Rehabilitation of World Federation of Chinese Medicine Societies and the Branch of Pulmonary Disease of the China Medical Association of Minorities organized experts to draw up Expert Consensus on Rehabilitation of Chinese Medicine for COVID-19 (First Edition) in light of the internationally recognized consensus-building method.^[18]^ The contents of the consensus include traditional exercise training such as shadowboxing, Tai-ji, Baduanjin Exercise, Qigong, or DAOYIN. It provides a reference for the rational selection and standard application of rehabilitation of Chinese medicine technology in the treatment of COVID-19. Pulmonary rehabilitation is difficult due to the high infectivity of COVID-19 and isolation of discharged patients. However, in recent years, the rapid development of Internet technology has provided the basis for remote monitoring and guidance. The use of information and communications technology in combination with wearable devices has made remote rehabilitation of patients with chronic lung disease possible, and its effectiveness and safety have been proven to be comparable to traditional methods.^[19,20]^ The traditional Chinese exercise (TCE) is simple in movement, and is not constrained by space concerns, has strong operability and implementability, which is conducive to a wide range of promotion. However, there is no direct evidence to show the efficacy of TCE on COVID-19 patients. This study aims to provide strong evidence support for the clinical practice of treating COVID-19 patients by conducting a systematic review and meta-analysis on the application of TCE in COVID-19 patients.

## 2 Methods

### 2.1 Study registration

This protocol was registered on PROSPERO platform (https://www.crd.york.ac.uk/prospero/display_record.php?ID=CRD42020179095), registration number: CRD42020179095. We have prepared this protocol in accordance with the Preferred Reporting Item for Systematic Review and Meta-analysis (PRISMA-P) statement,^[21]^ the anticipated start date of this study is December 01, 2020.

### 2.2 Ethics

Since meta-analysis does not involve the collection of private information, this research does not require ethical approval.

### 2.3 Eligibility criteria

Five main factors of PICOS were used for the review: participant (P), intervention (I), comparator (C), outcome (O), and study design (S).

### 2.4 Inclusion criteria

#### 2.4.1 Types of studies

Articles published in English or Chinese including:

Randomized Clinic Trials (RCTs) which investigated clinical efficacy and safety of TCE for COVID-19. No restrictions for blinding, follow-up, or publication status;

#### 2.4.2 Types of participants

1. Diagnosed as pneumonia caused by new coronavirus infection (2019-nCoV), light type and ordinary type patients;
2. Aged 18 to 70 years old;
3. Patients with clear consciousness;
4. Complete basic breathing training and physical exercise as required.

#### 2.4.3 Types of intervention and control

We will consider TCE involving shadowboxing, Baduanjin Exercise, Qigong, Taiji, DAOYIN, TENS, and combinations of these. There will be no restrictions for frequency, duration, or follow-up time of treatment.

#### 2.4.4 Types of comparators

Treatments in the comparison groups can be general treatment, pharmacotherapy, or no additional intervention to usual care.

#### 2.4.5 Types of outcome measures

We will screen clinical studies that report numerical data on one or more of the following outcomes:

##### 2.4.5.1 Primary outcomes

- Pulmonary function test: including the pulmonary ventilation function and diffusion function;
- Respiratory assessment: including the St Georges Respiratory Questionnaire and modified British Medical Research Council or other validated outcome measures;
- TCM symptom score.

##### 2.4.5.2 Secondary outcomes

- Pulse oxygen saturation (SpO_2_);
- 6-Minute walk test (6MWT);
- Results of chest X-ray or HRCT visual score;
- Quality of life measure by Rating of Perceived Exertion Scale, Short Form-36, Activities of daily living, or other outcomes;
- Psychological function assessment: including Self-rating Depression Scale, Self-rating Anxiety Scale, Patient Health Questionnaire-9, Generalized Anxiety Disorder Scale, Hamilton Depression Scale and Pittsburgh Sleep Quality Index (PSQI) or other outcomes;
- Incidence of adverse events.

If other outcomes are reported in the eligible studies, these will be extracted and reported but we will give particular attention to the possibility of selective reporting bias when using any such outcomes in our review.

### 2.5 Exclusion criteria

The exclusion criteria are as follows:

Incomplete data and duplicated data or data that cannot be extracted after contacting original authors.

The full text cannot be obtained after contacting the original authors.

### 2.6 Search strategy

We will search the following sources for the identification of trials: CNKI, VIP, WanFang Database, CBM, Medline, Embase, ISI, and CENTRAL. The time limit for literature retrieval is from the establishment of each database to 01, December 2020. The language is limited to English and Chinese. Search relevant journals and medical journals for potential non-electronic literature, and strict restrictions will be placed to exclude the types of studies that are not RCTs. Search terms will generally consist of 3 groups:

- Clinical conditions: novel coronavirus OR COVID-19 OR 2019-nCoV OR COVID-2019 pneumonia.
- Types of intervention: TCE such as shadowboxing, Baduanjin Exercise, Taiji, Qigong, DAOYIN.
- Study Type: Randomized Controlled Trial

According to the respective characteristics of the database, the comprehensive retrieval of the combined free words of the theme-word was carried out. The full search strategy for PubMed is provided in Table 1, and similar strategies will be applied to the other electronic databases. In addition, we will search for clinical trial registries, dissertations, and grey literature.

**Table 1:** Medline search strategy.

Number	Entry terms
#1	COVID-19 [Mesh]
#2	COVID-19 OR 2019 novel coronavirus infection OR 2019-nCoV infection OR COVID-19 pandemic OR coronavirus disease-19 OR 2019-nCoV disease OR COVID19 OR 2019 novel coronavirus disease OR coronavirus disease 2019 OR Coronavirus
#3	#1 OR #2
#4	Traditional Chinese Exercise [Mesh]
#5	Traditional Chinese Exercise OR Rehabilitation Exercise OR Exercise, Rehabilitation OR shadowboxing OR Tai chi OR Tai Ji Quan OR Taijiquan OR Tai-ji OR Baduanjin Exercise OR Qigong OR Chi Kung OR DAOYIN
#6	#4 OR #5
#7	Randomized Controlled Trial [Publication Type]
#8	Randomized Controlled Trial [Title/Abstract] OR models animal [Title/Abstract] OR models [Title/Abstract]
#9	#7 OR #8
#10	#3 AND #6 AND #9

### 2.7 Study selection and data extraction

**Figure 1:**
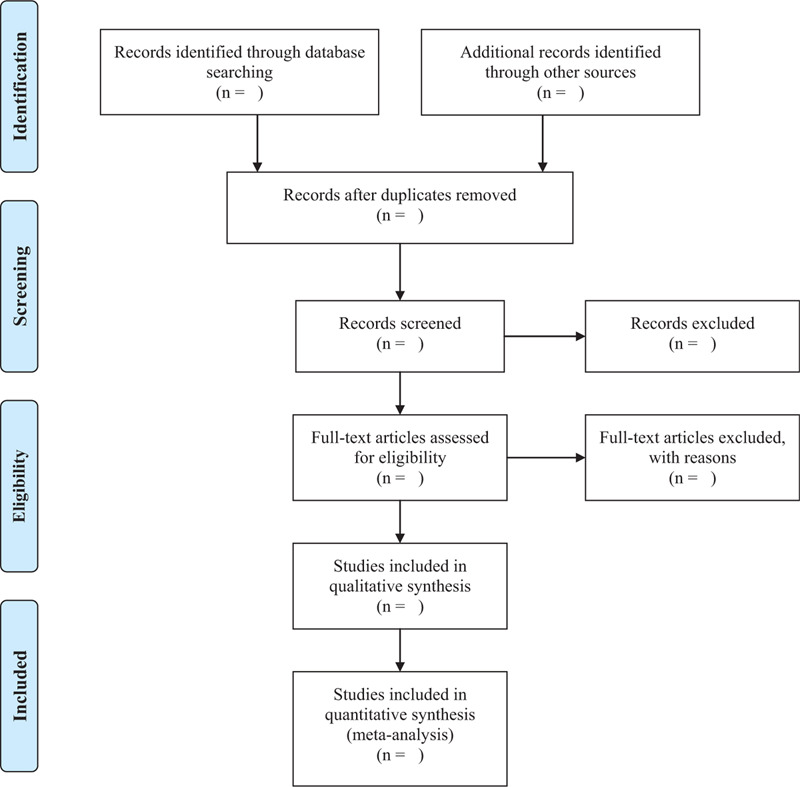
Flow chart of literature screening.

As shown in Figure 1, 2 reviewers will independently screen literature according to inclusion and exclusion criteria, eliminate the duplicate literature by EndNote X9.0, conduct a preliminary screening by reading the headline summary to exclude literature that does not meet the inclusion criteria, read the full text and making final selections, and data concerning details of study populations, interventions and outcomes will be extracted independently by 2 reviewers using a standard data extraction form. Any disagreement between them over the eligibility of particular studies will be resolved through discussion with a third reviewer. The standard data extraction form will include at least the following items: (a) Author details and year of publication; (b) Study design and recruitment strategy; (c) Sample size; (d) Method of diagnosis; (e) Participant demographics: age, gender, smoking status, comorbidities; (f) Laboratory findings; (g) Imaging findings; (h) Intervention characteristics: name, start date, frequency, and the length of time; (i) Rating scales: name, date, score; At the same time, the key factors of bias risk assessment are extracted. We will contact the corresponding authors for additional information if necessary.

### 2.8 Quality assessment

Two authors will use the Cochrane bias risk assessment tool^[22]^ to assess the risk of bias in trials based on 7 items: (a) random sequence generation (selection bias); (b) allocation concealment (selection bias); (c) blinding of participants and personnel (performance bias); (d) blinding of outcome assessment (detection bias); (e) incomplete outcome data (attrition bias); (f) selective reporting (reporting bias); (g) other sources of bias. Discrepancies in the interpretations will be resolved by consensus, or with the involvement of the corresponding author. For the above-mentioned 7 items, they were divided into 3 levels: low risk of bias, high risk of bias, and unclear risk of bias.

### 2.9 Evidence synthesis for RCTs

#### 2.9.1 Meta-analysis

This study will use RevMan.5.3 software for data analysis. Continuous data will be presented as mean differences with 95% confidence intervals (CIs), and dichotomous data will be presented as risk ratios with 95% CI and *P* values. The fixed-effects model will be employed if statistical heterogeneity was not significant (*I*^2^ < 50%). The random-effects model will be applied If the presence of substantial heterogeneity (*I*^2^ > 50%) in pooled studies.^[23]^ Subgroup analysis and sensitivity analysis will be performed for examining the potential causes.

#### 2.9.2 Subgroup analysis

When conducting meta-analysis, for each outcome studies will be grouped according to (a) the type of treatment (shadowboxing, Baduanjin Exercise, Qigong, Taiji, DAOYIN); (b) the comparator (no treatment, general treatment).

#### 2.9.3 Sensitivity analysis

Sensitivity analyses are planned based on clinical factors (age, comorbidities, Chinese medicine syndrome/pattern), intervention method (shadowboxing, Baduanjin Exercise, Qigong, Taiji, DAOYIN), methodological characteristics (sample size, risk of bias), and presence of statistical heterogeneity as applicable.

#### 2.9.4 Small sample effect/publication bias

We will assess publication bias using funnel plots for asymmetry when at least 10 trials are available.^[24]^ If the plot is asymmetric and there is no inverted funnel shape, it indicates that there may be publication bias. The reasons may be related to the small sample size, allocation concealment, and insufficient implementation of the blind method.

#### 2.9.5 Dealing with missing data

If the literature information is incorrect or incomplete, we will contact the first author via email address. If no response is received, the document should be deleted.

#### 2.9.6 Evaluating the quality of the evidence

To grade evidence quality and understand the current situation of evidence rating thereby analyzing possible problems, The Grading of Recommendations Assessment, Development, and Evaluation (GRADE) instrument will be used to assess the quality of evidence.^[25]^ Based on the bias, inconsistent, inaccurate, indirect, and the risk of publication bias 5 degradation factors, the quality classification for the 4 levels of evidence: high, medium, low, and very low.

## 3 Discussion

Since December 2019, Wuhan, China, has experienced an outbreak of COVID-19, caused by the severe acute respiratory syndrome coronavirus 2 (SARS-CoV-2). Under the background of the comprehensive medical treatment in China, the epidemic trend of COVID-19 gradually slowed down, and a large number of patients had been cured and discharged from hospital in China. During the period of the treatment, we found that the patients with COVID-19 suffer from varying degrees of respiratory, physical, and psychological dysfunction, it's important to gradually shift the emphasis of clinical work to COVID-19 convalescence therapy, especially pulmonary rehabilitation. TCE comprises comprehensive pulmonary rehabilitation interventions, including but not limited to respiratory rehabilitation, as well as psychological support, education, and behavioral changes. In TCM theoretical system, TCE is a mind-body training skill that can regulate the body, breath, and mind to improve physical function, and to prevent and treat diseases.^[26–28]^ TCE is characterized by smooth and gentle movement, which can achieve balance by adjusting body movement and posture. Regulation of breath involves changes in respiratory movement, rhythm, and pattern, it is beneficial to improve the lung function. Regulation of mind includes focusing attention and visualization. Most operations of mind regulation are similar to meditation,^[29–31]^ which can produce a relaxation response and stress reduction. TCE is often used in the prevention and treatment of respiratory infections.^[32]^ Considering the epidemic characteristics of the COVID-19, TCE has unique advantages for the movements are small and the space requirement is not significant. Thus, they are suitable for home practice during the current epidemic. With the rapid development of Internet technology, it has provided the basis for remote monitoring and guidance. The goal of it is not only to improve the patient's physical and mental conditions but also to help patients return to family and society more promptly.

In this study, based on the newly released Expert Consensus on Rehabilitation of Chinese Medicine for COVID-19, as well as the evidence from the clinical, we worked out a detailed plan intending to provide high-quality synthesis of the effects TCE for COVID-19. And this is the first systematic review and meta-analysis to examine empirical evidence of the application of TCE for COVID-19. It will provide an overview and assess the strengths and limitations of available evidence. However, there is a likely limitation that many articles published in Chinese are inadequate methodological reporting, second, the unified standards for clinical trial reporting and the standards for TCM reporting have not been adopted by many Chinese journals, so the information needed to assess the risk of bias may not be available in RCT reports,^[33–35]^ which may lead to the risk of bias.

## Author contributions

**Conceptualization:** Yuanyuan Duan, Mengran Xiong, Xiaoyan Yao.

**Data curation:** Yuanyuan Duan, Mengran Xiong, Hengyuan Liu.

**Formal analysis:** Hengyuan Liu, Heping Wang.

**Funding acquisition:** Xiaoyan Yao, Guangxi Li.

**Methodology:** Yuanyuan Duan, Heping Wang, Guangxi Li.

**Project administration:** Xiaoyan Yao, Heping Wang, Hengyuan Liu.

**Writing – original draft:** Yuanyuan Duan, Mengran Xiong, Heping Wang

**Writing – review & editing:** Guangxi Li.
